# Alcohol-Related Physical Abuse of Children in the Slums of Kampala, Uganda

**DOI:** 10.3390/ijerph14101124

**Published:** 2017-09-26

**Authors:** Monica H. Swahn, Rachel E. Culbreth, Catherine A. Staton, Shannon R. Self-Brown, Rogers Kasirye

**Affiliations:** 1 School of Public Health, Georgia State University, P.O. Box 3984, Atlanta, GA 30302, USA; rculbreth@student.gsu.edu (R.E.C.); sselfbrown@gsu.edu (S.R.S.-B.); 2 Duke University Medical Center, Duke Global Health Institute and Department of Emergency Medicine, Duke University, Durham, NC 27703, USA; Catherine.staton@duke.edu; 3 Uganda Youth Development Link, Sir Appollo Kaggwa Rd, Box 12659, Kampala, Uganda; kasiryer@yahoo.com

**Keywords:** international health, child physical abuse and neglect, alcohol use, alcohol use initiation, urban, slums

## Abstract

This study examines the patterns of alcohol-related physical abuse and alcohol use and related behaviors among children living in the slums of Kampala, Uganda. The study is based on a cross-sectional survey, conducted in spring 2014, of service-seeking children ages 12 to 18 years (*n* = 1134) attending Uganda Youth Development Link drop-in centers for vulnerable children in the slums. Descriptive statistics, chi-squares, and bivariate and multivariable logistic regression analyses were conducted to determine patterns of children’s alcohol-related behaviors, based on alcohol-related physical abuse and neglect. Nearly 34% of children (*n* = 380) reported experiencing physical abuse, and 12.4% (*n* = 140) reported experiencing alcohol-related physical abuse. Alcohol-related neglect was reported among 19.6% (*n* = 212) of the children. Past year alcohol use was significantly more prevalent among children who reported experiencing alcohol-related neglect ($\chi^{2}$ = 79.18, *df* = 1, *p* < 0.0001) and alcohol-related physical abuse ($\chi^{2}$ = 62.02, *df* = 1, *p* < 0.0001). Reporting physical abuse was also associated with parental alcohol use (OR: 1.85; 95% CI: 1.38, 2.48) and parental partner violence (OR: 5.51; 95% CI: 4.09, 7.43), after adjusting for other variables in the model. Given the high levels of alcohol-related abuse and neglect reported in this population, both primary and secondary prevention initiatives are needed to improve parenting strategies and to reduce alcohol-related harm. Similarly, strategies to reduce and delay alcohol use among these vulnerable children are also needed.

## 1. Introduction

Child abuse and neglect is a global concern with devastating consequences, including poor school performance, chronic diseases, HIV, mental health issues, injuries and suicide [1,2,3,4]. In 2014 alone, an estimated one billion children globally were exposed to child abuse and violence [1]. In a recent systematic review, Africa was identified to have among the highest levels of child exposure to overall violence with a past-year prevalence of 50% [1]. Other estimates of physical and sexual child abuse in Africa are very high, at 64% [5,6]. Research has shown that the burden of child abuse and neglect, in terms of prevalence and economic burden, is the heaviest in low- and middle-income countries [7]. However, there are few empirical studies examining this issue in sub-Saharan Africa.

Parental or caregiver alcohol misuse is a major risk factor for child abuse and neglect [8,9]. A study in South Africa reports parental alcohol and drug problems are associated with child physical abuse specifically [10]. Another study conducted in Alexandria, Egypt, reports similar associations between parental alcohol use and child physical abuse [11].

In the broader context of child abuse and neglect, whether fueled by alcohol or not, researchers have identified several risk and protective factors at the child level, parental and caregiver level, and on the social and environmental level. At the child level, factors associated with experiencing child abuse and neglect include age [9,10,11], childhood trauma [9], such as ever being raped, and gender [9]. Conflicting reports of the association with gender and child physical abuse exist in the literature, with some reporting that females are more likely to experience abuse [9] and others reporting that males are more likely to experience abuse [5]. Child protective factors include good health [9], positive peer relationships [9], and exhibiting a positive outlook on the future, specifically when the abuser is no longer present [12]. At the parental level, in addition to alcohol and substance misuse, factors associated with child abuse and neglect include domestic violence [5,9], high general stress level, social isolation, and poor parent-child interaction [9]. Social and environmental risk factors for child abuse and neglect include low socioeconomic status, stressful life events, homelessness, and living in a dangerous/violent neighborhood [9].

Consequently, children who experience parental abuse are at a high risk for engaging in substance use behaviors, including alcohol use [5]. Childhood physical abuse and neglect has been linked to heavy episodic drinking in adolescents and young adults [13]. The consequences of experiencing child maltreatment, including early alcohol use initiation, may be more severe in low- and middle-income countries, where resources and infrastructure to support those who have experiences child maltreatment are scarce [5,14]. Additionally, children whose parents misuse alcohol are themselves more likely to consume alcohol [15,16,17]. This cyclical nature of parental alcohol consumption has been linked to an earlier onset of alcohol use among children, as well as alcohol use disorders throughout adolescence and adulthood [15]. Persistent family history of alcohol misuse is also associated with heavy alcohol consumption throughout adolescence and adulthood [15]. Moreover, parental violence, whether domestic towards a partner or towards the child, also exerts a cyclical relationship of violence for the child. Children who experience parental abuse or witness parental interpersonal violence are at a much higher risk of becoming a perpetrator and victim of violence later in life [17]. Therefore, adults who engage in heavy alcohol consumption and additionally commit child abuse and neglect are exhibiting multiple mechanisms of influence on their children and increasing their risk of alcohol misuse [15,16,17].

Despite substantial evidence of alcohol-related child abuse and neglect in some parts of the world, research is scarce on this topic among children in sub-Saharan Africa, especially among the most vulnerable children who live in the slums. Children who live in the slums of Kampala, Uganda are at a heightened risk of experiencing maltreatment due to the dire living conditions of the slums, including high rates of substance use and familiar, interpersonal, and community violence [18,19,20,21,22], food scarcity [23], high unemployment, and lack of parental oversight [18,19,20,21,22].

In order to examine the prevalence and context for alcohol-related abuse and neglect in this population we formulated the following research questions: (1) What are the characteristics of children who report any physical abuse and any alcohol-related abuse?; (2) What are the associations between perceived parental alcohol use, lifetime alcohol-related neglect and lifetime alcohol-related abuse?; (3) What are the alcohol use behaviors and help-seeking behaviors among children who report parental alcohol-related neglect and alcohol-related physical abuse? and (4) What are the child-level, parent-level, and environmental-level correlates of overall lifetime exposure to physical child abuse? The purpose of this paper is to determine patterns of alcohol-related behavior and of physical abuse, in order to inform primary and secondary prevention efforts for child abuse and potential consequences of child abuse, including alcohol use among service-seeking youth. Please note that for the purposes of this paper, the terms “child abuse and neglect” as defined by the WHO, encompass a broad definition of physical and emotional abuse and neglect that negatively affects the youth’s “health, development, or dignity” [24].

## 2. Materials and Methods

### 2.1. Setting

The Kampala Youth Survey was a cross-sectional study conducted in spring of 2014 to examine risk behaviors and exposures among children living in the slums of Kampala. The primary focus of the survey was to assess alcohol use, violence, sexual risk behaviors and HIV. At the time the study was conducted, the children were participating in a Uganda Youth Development Link (UYDEL) drop-in center and were 12–18 years of age [22]. The urban UYDEL centers provide vocational training, sexual health services, and mental health counseling for disadvantaged youth who are living on the streets or in the slums of Kampala [25]. Youth who attended UYDEL drop-in centers were recruited while participating in various activities or while engaging in community outreach activities in neighborhoods surrounding the drop-in centers [22]. At the time of this study, UYDEL provided services to about 650 youth per month across the six urban centers.

### 2.2. Data Collection

Over the 14-day data collection period, 1628 children were asked to participate in the survey. Among these, 92% agreed to participate (131 declined), yielding a total of 1497 surveys collected. Several surveys were lost due to technical software issues (320), and therefore the final sample consisted of 1134 surveys [22]. The survey was administered to the participants on Google Nexus 7 tablets using the Qualtrics survey software (Qualtrics, Provo, UT, USA). The use of tablets as an mHealth technology allowed for easier administration of the survey and streamlined data collection. UYDEL interviewers received a one-day training on survey administration protocols and the use of the tablet [22].

Survey questions were translated into Luganda (the most common local language in the region) by a certified translator and back-translated for accuracy so that the survey could be offered in both English and Luganda. In-person interviews lasting 20–30 min were conducted by UYDEL staff after proper consent procedures (parental consent had been waived for youth ages 12–17) [22]. While sensitive topics were covered in the survey, the parental consent waiver was approved for three reasons: (1) In Uganda, children who cater to their own livelihood are considered emancipated at age 14; (2) Children as young as 12 can consent to HIV testing without parental consent; and (3) Because many of these youth are orphans and otherwise “abandoned”, UYDEL is considered serving the children and their best interest. Participation was limited to youth ages 12–18. There were no exclusion criteria. Youth recruited received a small snack and a drink (juice or soda) as incentive for participating in the survey [22]. All subjects gave their informed consent for inclusion before they participated in the study [22]. The study was conducted in accordance with the Declaration of Helsinki, and the protocol was approved by the Institutional Review Board at Georgia State University (H14101) and the Uganda National Council on Science and Technology (SS3338).

The questions included in the Kampala Youth Survey 2014 were mostly collected from previously validated instruments, including: Global School-based Student Health Survey (GSHS) [26], Kampala Youth Survey 2011 [18,19,20,21,22], MAMPA 2012 Questionnaire, AUDIT Questionnaire [27], CAGE Questionnaire [28], iMPPACS, AIDS Indicator Survey [29], and the Demographic Health Survey [30].

### 2.3. Measures

The main outcomes examined in the current paper include child-reported lifetime physical abuse, child-reported lifetime alcohol-related physical abuse, and child-reported lifetime alcohol-related neglect, all of which were each one-item measures and asked about abuse and neglect by their parents. Child physical abuse was measured by one question: “Did your parents ever beat you so hard you had bruises or marks?”. Alcohol-related child physical abuse was measured using one question: “Did a parent beat you when they were drunk?”. Finally, alcohol-related child neglect was measured using one question: “Did your parent’s/caretaker’s alcohol use make them not able to care for you?”. Alcohol use behaviors and problem drinking were assessed among those reporting physical abuse, alcohol-related physical abuse, and alcohol-related neglect. Problem drinking was assessed using multiple measures of binge drinking, alcohol frequency, and CAGE scores [31].

The predictors for the physical abuse bivariate and multivariable logistic regression analyses include gender (male/female), age, ever being raped, feeling hopeful about the future, overall health, parental alcohol use, parental partner violence, ever living on the streets and living in an unsafe neighborhood. Ever being raped was measured using, “Has someone ever raped you or forced you to have sex with him or her?”. Participants could answer “Yes” or “No”. Feeling hopeful about the future was measured using, “In the past month, how often did you feel hopeful about the future?”. Participants could answer “Never”, “Sometimes” and “Often”. Overall health was measured using, “How would you rate your physical health?”. Participants could answer “Excellent”, “Good”, “Fair”, and “Poor”. Parental alcohol use was measured using, “Did your parents/caretakers drink a lot of alcohol when you were growing up?”. Participants could answer “Yes” or “No”. Parental partner violence and parental domestic violence was measured using, “Did you ever see or hear your parents beating each other?”. Participants could answer “Yes” or “No”. Ever living on the streets was measured using, “Have you ever lived on the streets with no other place to go?”. Participants could answer “Yes” or “No”. Living in an unsafe neighborhood was measured using, “Overall what do you think about the following statement—I feel safe in this neighborhood”. Participants could answer “Yes” or “No” and answers were coded so that the higher response value of 1 corresponded to “No” or living in an unsafe neighborhood.

### 2.4. Data Analysis

Descriptive statistics were computed for characteristics among children who reported physical abuse, alcohol-related physical abuse, and alcohol-related neglect. Alcohol use behaviors and problem drinking were examined among children who reported alcohol-related physical abuse and alcohol-related neglect. Chi-Square tests were conducted to determine differences of these behaviors and characteristics between children who reported abuse compared to children who did not report abuse. Bivariate and multivariable associations were computed using logistic regression analyses to determine the associated correlates with experiencing physical abuse overall. All analyses were conducted in SAS 9.4 (SAS Institute Inc., Cary, NC, USA).

## 3. Results

Demographic characteristics of children who reported any physical abuse and alcohol-related physical abuse are presented in Table 1. Nearly 34% of children (*n* = 380) reported experiencing physical abuse, and 12.4% (*n* = 140) reported experiencing alcohol-related physical abuse. There were no statistically significant differences in gender, age, education, or parental living status among children who reported physical abuse. There was a statistically significant difference among children who reported physical abuse and ever living on the streets ($\chi^{2}$ = 16.19, *df* = 1, *p* < 0.0001). A higher percentage of children who reported physical abuse also reported ever living on the streets compared to children who did not report physical abuse (29.0% vs. 18.5%, respectively). Among children who reported alcohol-related physical abuse, there were statistically significant differences in gender, education, parental living status, and ever living on the streets. A higher percentage of males reported experiencing alcohol-related physical abuse compared to not experiencing alcohol-related physical abuse (51.4% vs. 42.8%, respectively).

There was also substantial overlap of the different types of abuse and neglect experienced. Among youth who reported any physical abuse (*n* = 380), 29.8% (*n* = 113) also reported experiencing alcohol-related physical abuse. Additionally, among youth who reported any physical abuse, 30.8% (*n* = 117) also reported alcohol-related neglect. Among the total sample, 6.5% (*n* = 74) reported experiencing both alcohol-related abuse and neglect. Moreover, 5.7% (*n* = 65) reported physical abuse, alcohol-related physical abuse, and alcohol-related neglect.

Alcohol use perceptions and alcohol-related behaviors among children who reported alcohol-related neglect and alcohol-related physical abuse are presented in Table 2. Alcohol-related neglect was reported among 19.6% of children (*n* = 212). A significantly higher percentage of children reported parental approval of alcohol use among those who reported alcohol-related neglect compared to those who reported no neglect (35.6% vs. 12.4%, respectively ($\chi^{2}$ = 64.75, *df* = 1, *p* < 0.0001). Similar findings existed among children who reported any physical abuse ($\chi^{2}$ = 45.65, *df* = 1, *p* < 0.0001) and alcohol-related physical abuse ($\chi^{2}$ = 70.42, *df* = 1, *p* < 0.0001). There was also a statistically significant difference in alcohol use among children who reported physical abuse ($\chi^{2}$ = 42.18, *df* = 1, *p* < 0.0001), alcohol-related neglect ($\chi^{2}$ = 79.18, *df* = 1, *p* < 0.0001) and alcohol-related physical abuse ($\chi^{2}$ = 62.02, *df* = 1, *p* < 0.0001). Prevalence of past year alcohol use was significantly higher among children reporting alcohol-related neglect (56.6%) and alcohol-related physical abuse (59.3%) compared to children who did not reported alcohol-related neglect (25.0%) or alcohol-related physical abuse (26.5%). There was also a statistically significant difference in age of alcohol initiation for physical abuse ($\chi^{2}$ = 43.61, *df* = 3, *p* < 0.0001), alcohol-related neglect ($\chi^{2}$ = 85.38, *df* = 3, *p* < 0.0001) and alcohol-related physical abuse ($\chi^{2}$ = 80.16, *df* = 3, *p* < 0.0001). No statistically significant differences were found for frequency of alcohol use, frequency of full drinks consumed, or binge drinking (consuming five or more drinks on one occasion) for any type of abuse. Children who reported alcohol-related neglect reported a higher prevalence of days with a hangover (80.0%) compared to children who did not report alcohol-related neglect (65.1%) ($\chi^{2}$ = 8.21, *df* = 1, *p* = 0.004). There was a statistically significant difference in problem drinking, as defined by CAGE scores, for alcohol-related neglect ($\chi^{2}$ = 6.04, *df* = 1, *p* = 0.01). However, this finding was not statistically significant for alcohol-related physical abuse nor parental physical abuse.

Bivariate and multivariable associations of physical abuse and alcohol-related physical abuse are displayed in Table 3. In the multivariable model, experiencing physical abuse was associated with parental alcohol use (OR: 1.85; 95% CI: 1.38, 2.48) and parental partner violence (OR: 5.51; 95% CI: 4.09, 7.43), after adjusting for the other variables in the model. For the bivariate associations, physical abuse was associated with the child report of having been raped (OR: 1.53; 95% CI: 1.11, 2.10), overall worse health (poor vs. excellent health, OR: 2.23; 95% CI: 1.28, 3.89), parental alcohol use (OR: 2.91; 95% CI: 2.25, 3.76), parental partner violence (OR: 6.67; 95% CI: 5.03, 8.85), ever living on the streets (OR: 1.80; 95% CI: 1.35, 2.40), and living in an unsafe neighborhood (OR: 1.63; 95% CI: 1.22, 2.17). In the multivariable model for alcohol-related parental physical abuse, statistically significant correlates include being female (OR: 0.59; 95% CI: 0.39, 90), age (OR: 1.16; 95% CI: 1.03, 1.31), ever being raped (OR: 1.69; 95% CI: 1.03, 2.77), parental partner violence (OR: 7.51; 95% CI: 5.01, 11.25), and living in an unsafe neighborhood (OR: 1.62; 95% CI: 1.06, 2.48). For the bivariate associations, alcohol-related parental physical abuse was associated with age (OR: 1.16; 95% CI: 1.04, 1.29), ever being raped (OR: 2.03; 95% CI: 1.35, 3.06), overall worse health (poor vs. excellent health, OR: 2.74; 95% CI: 1.30, 5.80), parental partner violence (OR: 7.82; 95% CI: 5.29, 11.56), ever living on the streets (OR: 1.76; 95% CI: 1.19, 2.60), and living in an unsafe neighborhood (OR: 2.15; 95% CI: 1.47 3.13).

## 4. Discussion

This paper presents new and empirical findings on physical abuse, alcohol-related physical abuse, and alcohol-related neglect among children living in the slums of Kampala, Uganda. This population is particularly vulnerable due to many extenuating circumstances and environmental conditions [18,19,20,21,22]. Our findings show that nearly one in three of the children reported experiencing physical abuse. Additionally, a large percentage of children also reported alcohol-related abuse and neglect. Parenting interventions and prevention strategies to reduce injuries and adverse outcomes related to physical child abuse and alcohol use is urgently warranted in this population. 

Our findings are similar to previous studies conducted elsewhere with regards to the association between parental alcohol-related physical abuse and neglect with children’s own alcohol-related behaviors [15,16,17]. In our study, children who reported physical abuse and neglect also reported a higher prevalence of past-year alcohol use and an earlier age of alcohol use initiation. Additionally, a higher percentage of children who experienced both alcohol-related physical abuse and neglect also reported a higher prevalence of parental approval of or apathy about child alcohol use. Future studies should examine the potentially mediating and/or moderating role of parental approval of alcohol use on the association between alcohol-related parental harm and children’s own alcohol use.

In our more conservative multivariable logistic regression model, parental factors were the only correlates associated with overall parental physical abuse. Previous studies also show that parental alcohol use and parental partner violence are strongly linked with physical child abuse and neglect [5,9]. We did not detect any statistically significant association in the final multivariable model with child-level factors (age, gender, previously being raped, hopeful outlook on the future, and overall health) or social/environmental factors (previously living on the streets and living in an unsafe neighborhood). While some of these associations were statistically significant in the bivariate associations, in the final multivariable model, the only correlates that remained statistically significant after adjusting for other variables were the parent-level factors. 

### Limitations

Due to the cross-sectional nature of this study, causal mechanisms cannot be assumed or inferred. Additionally, the timeframe of the multiple variables and outcomes examined may overlap and therefore make it impossible to establish the sequencing and timeline of these experiences. Specifically, the timeframe and context of abuse were not assessed in this survey since the primary goal was to determine alcohol-related behaviors, alcohol marketing, and HIV/sexual risk behaviors. Accordingly, these findings should be interpreted as merely correlates of physical abuse, alcohol-related physical abuse, and alcohol-related neglect. Due to the sensitive topics discussed, social desirability bias and misclassification may also be present and have likely produced an underestimate of the overall and true prevalence of abuse and neglect in this population. Moreover, the convenience sample of children surveyed is also limiting; however, it should be noted that this population is hard-to-reach and a clear sampling frame does not exist. These limitations are mitigated by the scarcity of empirical findings in this and similar populations related to abuse and neglect. Additionally, the findings can also be used to generate support for more in-depth research of this understudied topic.

## 5. Conclusions

Despite these limitations, this is the first paper, to our knowledge, that quantifies the prevalence of physical abuse, alcohol-related physical abuse, and alcohol-related neglect among youth living in the slums of Kampala, Uganda and in the broader region. Our findings clearly demonstrate increased levels of alcohol use and alcohol-related behavior among children who report abuse and neglect. Future studies should further examine the associations of alcohol-related physical abuse and neglect in this and similar populations to better understand the context as well as the causal factors including any potential mediating and moderating effects that can be targeted in prevention strategies. Future research should also examine the structural drivers that may influence alcohol use trajectories among these children while also assessing exposure to alcohol harm within the family and the extended family and social networks. This may shed light on the driving influences behind early alcohol use among children living in the slums of Kampala, where structural drivers of alcohol use such as marketing is surging and regulation of alcohol marketing is lacking [32,33,34,35].

As recommended by the World Health Organization, interventions to prevent child abuse and neglect should focus on the development of stable relationships between parents and children and are urgently warranted in this population [36]. In one of the few reviews of parenting programs implemented in low- and middle-income countries, findings suggest that parental behaviors can improve in these contexts, however, very few of the studies actually measure future violence or maltreatment as an outcome [37]. However, there is increasing support for parenting programs that effectively reduce maltreatment risk and reoccurrence in high resource countries, such as the U.S. [38,39]. Even so, in high-income countries, there is also limited research examining the impact of integrated parenting intervention and substance abuse treatment for parents, which are imperative based on the findings in this current study [40]. The one exception is emerging work by Donohue and colleagues which is examining the impact of Family Behavior Therapy in addressing these multiple risk factors in child welfare involved families in the U.S. [41]. Further work is needed to understand how evidence-based programming can be adapted and implemented in low resource countries, especially programs that directly address the costs involved to build the infrastructure and train professionals to effectively deliver such programs.

The SASA! Study in Uganda previously tested an intervention to decrease intimate partner violence and children’s exposure to violence in the home [42]. That intervention emphasized gender equality and the prevention of violence through a variety of mediums, focusing primarily on healthy communication and relationship skills [42]. The SASA! Study reported a 52% reduction in women’s exposure to physical violence and a 64% reduction in children’s exposure to intimate partner violence in the home [43]. Future research should investigate the feasibility of implementing this intervention, or a tailored version of the intervention, among families living in the slums to also specifically target reduction of alcohol-related harm.

Future research should also identify strategies to prevent early alcohol initiation among those who experience child abuse. Evidence based interventions addressing alcohol use at the community (media and alcohol availability) [44,45,46], family [47], and individual level (brief interventions) [48] have had limited implementation or evaluations in a Sub-Saharan African setting; further cultural adaptation and implementation of these multi-level interventions is warranted [49]. Reducing the violence and alcohol use among the children would greatly decrease their high risk exposures with an ultimate goal of subsequently decreasing adverse health outcomes and the often cyclical relationship of alcohol use and violence.

## Figures and Tables

**Table 1:** Demographic characteristics of children who reported any parental physical abuse or any alcohol-related physical abuse among youth living in the slums of Kampala, (*n* = 1134).

Variables	Physical Abuse			Alcohol-Related Physical Abuse		
	Yes (*n* = 380) 33.7%	No (*n* = 749) 66.3%	Chi-Square, (*df*), *p*-Value	Yes (*n* = 140) 12.4%	No (*n* = 988) 87.6%	Chi-Square, (*df*), *p*-Value
Gender						
Male	168 (44.2%)	326 (43.6%)	0.04, (*1*), *p* = 0.84	72 (51.4%)	422 (42.8%)	3.75, (*1*), *p* = 0.05
Female	212 (55.8%)	422 (56.4%)		68 (48.6%)	565 (57.2%)	
Age, Mean (SD)	16.3 (1.7)	16.0 (1.8)		16.5 (1.5)	16.1 (1.8)	
Education						
Primary or less	130 (34.4%)	266 (36.1%)	3.39, (*2*), *p* = 0.18	37 (26.4%)	359 (36.9%)	6.81, (*2*), *p* = 0.03
Completed primary	80 (21.2%)	183 (24.8%)		42 (30.0%)	221 (22.7%)	
Secondary or higher	168 (44.4%)	288 (39.1%)		61 (43.6%)	394 (40.5%)	
Parental living status						
Both parents dead	96 (25.3%)	154 (20.6%)	3.34, (*2*), *p* = 0.19	42 (30.0%)	208 (21.1%)	7.21, (*2*), *p* = 0.03
One parent dead	139 (36.6%)	284 (37.9%)		53 (37.9%)	368 (37.3%)	
Both parents living	145 (38.2%)	311 (41.5%)		45 (32.1%)	412 (41.7%)	
Ever lived on the streets with nowhere to go						
Yes	110 (29.0%)	138 (18.5%)	16.19, (*1*), *p* < 0.0001	44 (31.4%)	204 (20.7%)	8.27, (*1*), *p* = 0.004
No	270 (71.0%)	610 (81.5%)		96 (68.6%)	783 (79.3%)	

**Table 2:** Alcohol use perceptions, behaviors and help-seeking among youth living in the slums of Kampala reporting any physical abuse, alcohol-related neglect or alcohol-related physical abuse by their parents, (*n* = 1134).

Variables	Physical Abuse			Alcohol-Related Neglect			Alcohol-Related Physical Abuse		
	Yes (*n* = 380) 33.7%	No (*n* = 749) 66.3%	Chi-Square, (*df*), *p*-Value	Yes (*n* = 212) 19.6%	No (*n* = 870) 80.4%	Chi-Square, (*df*), *p*-Value	Yes (*n* = 140) 12.4%	No (*n* = 988) 87.6%	Chi-Square, (*df*), *p*-Value
Parental Approval of Child Alcohol Use									
Approve/Not Care	101 (26.7%)	82 (11.0%)	45.65, (*1*), *p* < 0.0001	75 (35.6%)	108 (12.4%)	64.75, (*1*), *p* < 0.0001	57 (40.7%)	126 (12.8%)	70.42, (*1*), *p* < 0.0001
Disapprove	278 (73.4%)	667 (89.0%)		136 (64.4%)	763 (87.6%)		83 (59.3%)	861 (87.2%)	
Alcohol use in the past year									
Yes	164 (43.2%)	182 (24.3%)	42.18, (*1*), *p* < 0.0001	120 (56.6%)	218 (25.0%)	79.18, (*1*), *p* < 0.0001	83 (59.3%)	262 (26.5%)	62.02, (*1*), *p* < 0.0001
No	216 (56.8%)	567 (72.7%)		92 (43.4%)	653 (75.0%)		57 (40.7%)	726 (73.5%)	
Age at consumption of first alcoholic beverage									
<13	28 (7.4%)	38 (5.1%)	43.61, (*3*), *p* < 0.0001	23 (11.0%)	34 (3.9%)	85.38, (*3*), *p* < 0.0001	22 (15.8%)	36 (3.7%)	80.16, (*3*), *p* < 0.0001
13–16	125 (33.1%)	156 (21.0%)		95 (45.2%)	177 (20.4%)		61 (43.9%)	219 (22.3%)	
>16	32 (8.5%)	26 (3.5%)		14 (6.7%)	51 (5.9%)		9 (6.5%)	57 (5.8%)	
Non-drinker+	193 (51.1%)	524 (70.4%)		78 (37.1%)	604 (69.8%)		47 (33.8%)	670 (68.2%)	
Who usually gives you alcohol to drink									
A family member/relative	10 (6.1%)	15 (8.2%)	7.37, (*4*), *p* = 0.12	4 (3.3%)	21 (9.6%)	7.73, (*4*), *p* = 0.10	7 (8.4%)	18 (6.8%)	11.83, (*4*), *p* = 0.02
A friend	75 (45.5%)	87 (47.8%)		51 (42.5%)	106 (48.4%)		30 (36.1%)	131 (49.8%)	
A sex partner	14 (8.5%)	23 (12.6%)		17 (14.2%)	20 (9.1%)		5 (6.0%)	32 (12.2%)	
I get it myself	65 (39.4%)	52 (28.6%)		46 (38.3%)	68 (31.1%)		40 (48.2%)	77 (29.3%)	
Other	1 (0.6%)	5 (2.8%)		2 (1.7%)	4 (1.8%)		1 (1.2%)	5 (1.9%)	
Age at first drunkenness									
<13	8 (4.9%)	15 (8.2%)	4.14, (*3*), *p* = 0.25	12 (10.0%)	11 (5.0%)	7.59, (*3*), *p* = 0.06	6 (7.2%)	17 (6.5%)	3.21, (*3*), *p* = 0.36
13–16	104 (63.0%)	102 (56.0%)		77 (64.2%)	123 (56.2%)		55 (66.3%)	150 (57.0%)	
>16	29 (17.6%)	43 (23.6%)		20 (16.7%)	52 (23.7%)		12 (14.5%)	60 (22.8%)	
Never/Non-drinker	24 (14.6%)	22 (12.1%)		11 (9.2%)	33 (15.1%)		10 (12.0%)	36 (13.7%)	
Whom do you usually drink alcohol with?									
With my friends	120 (72.7%)	133 (73.1%)	2.77, (*2*), *p* = 0.25	84 (70.0%)	161 (73.5%)	1.28, (*2*), *p* = 0.53	58 (69.9%)	195 (74.1%)	6.96, (*2*), *p* = 0.03
With others (i.e., family, sex partner)	30 (18.2%)	40 (22.0%)		25 (20.8%)	45 (20.6%)		14 (16.9%)	55 (20.9%)	
I drink alone	15 (9.1%)	9 (5.0%)		11 (9.2%)	13 (5.9%)		11 (13.3%)	13 (4.9%)	
Frequency of alcoholic beverage consumption									
Monthly or less	35 (21.2%)	35 (19.3%)	0.74, (*2*), *p* = 0.69	26 (21.7%)	42 (19.3%)	5.66, (*2*), *p* = 0.06	16 (19.3%)	54 (20.6%)	1.66, (*2*), *p* = 0.44
2–4 times a month	46 (27.9%)	58 (32.0%)		27 (22.5%)	76 (34.9%)		21 (25.3%)	83 (31.7%)	
5 or more times a month	84 (50.9%)	88 (48.6%)		67 (55.8%)	100 (45.9%)		46 (55.4%)	125 (47.7%)	
Frequency of full drinks consumed in typical day when drinking alcohol									
1–2 drinks	99 (60.0%)	96 (53.3%)	1.56, (*1*), *p* = 0.21	64 (53.3%)	124 (57.1%)	0.45, (*1*), *p* = 0.50	49 (59.0%)	146 (55.9%)	0.25, (*1*), *p* = 0.62
3 or more drinks	66 (40.0%)	84 (46.7%)		56 (46.7%)	93 (42.9%)		34 (41.0%)	115 (44.1%)	
Days of having a hangover, feeling sick, getting into trouble with friends or family, miss school, or get into fights because of drinking alcohol?									
0 days	45 (27.3%)	59 (32.6%)	1.16, (*1*), *p* = 0.28	24 (20.0%)	76 (34.9%)	8.21, (*1*), *p* = 0.004	19 (22.9%)	85 (32.4%)	2.73, (*1*), *p* = 0.10
1 or more days	120 (72.7%)	122 (67.4%)		96 (80.0%)	142 (65.1%)		64 (77.1%)	177 (67.6%)	
How many times (if any) have you had five or more drinks on one occasion?									
0 days	50 (30.3%)	53 (29.4%)	0.03, (*1*), *p* = 0.86	29 (24.2%)	71 (32.7%)	2.71, (*1*), *p* = 0.10	23 (27.7%)	80 (30.7%)	0.26, (*1*), *p* = 0.61
1 or more days	115 (69.7%)	127 (70.6%)		91 (75.8%)	146 (67.3%)		60 (72.3%)	181 (69.4%)	
Problem drinkers (as defined by CAGE Scores)									
Non-problem drinker (CAGE score 0–1)	81 (49.7%)	99 (54.7%)	0.86, (*1*), *p* = 0.35	51 (43.5%)	122 (56.5%)	6.04, (*1*), *p* = 0.01	43 (51.8%)	136 (52.3%)	0.006, (*1*), *p* = 0.94
Problem drinker (CAGE score ≥2)	82 (50.3%)	82 (45.3%)		69 (57.5%)	94 (43.5%)		40 (48.2%)	124 (47.7%)	

**Table 3:** Correlates of experiencing parental physical abuse and alcohol-related physical abuse among children living in the slums of Kampala, (*n* = 1134).

Independent Variables	Physical Abuse		Alcohol-Related Physical Abuse	
	Unadjusted Odds Ratios (95% CI)	Adjusted Odds Ratios (95% CI)	Unadjusted Odds Ratios (95% CI)	Adjusted Odds Ratios (95% CI)
*Childhood level factors*				
Gender				
Female	0.98 (0.76, 1.25)	0.99 (0.74, 1.33)	0.71 (0.50, 1.01)	0.59 (0.39, 0.90)
Male	1.00	1.00	1.00	1.00
Age	1.09 (1.01, 1.17)	1.06 (0.97, 1.15)	1.16 (1.04, 1.29)	1.16 (1.03, 1.31)
Ever being raped				
Yes	1.53 (1.11, 2.10)	1.11 (0.75, 1.63)	2.03 (1.35, 3.06)	1.69 (1.03, 2.77)
No	1.00	1.00	1.00	1.00
Feeling hopeful about the future				
Never	1.00	1.00	1.00	1.00
Sometimes	0.95 (0.64, 1.42)	1.06 (0.67, 1.69)	1.06 (0.61, 1.86)	1.14 (0.61, 2.12)
Often	0.94 (0.63, 1.40)	0.98 (0.62, 1.55)	0.78 (0.44, 1.37)	0.76 (0.41, 1.44)
Overall health				
Excellent	1.00	1.00	1.00	1.00
Good	0.95 (0.67, 1.34)	0.55 (0.29, 1.07)	1.32 (0.77, 2.27)	1.53 (0.85, 2.76)
Fair	1.52 (1.03, 2.25)	0.56 (0.31, 1.01)	1.74 (0.96, 3.17)	1.83 (0.94, 3.58)
Poor	2.23 (1.28, 3.89)	0.87 (0.47, 1.61)	2.74 (1.30, 5.80)	2.31 (0.99, 5.43)
*Parental Factors*				
Alcohol use				
Yes	2.91 (2.25, 3.76)	1.85 (1.38, 2.48)	---	
No	1.00	1.00		---
Parental partner violence				
Yes	6.67 (5.03, 8.85)	5.51 (4.09, 7.43)	7.82 (5.29, 11.56)	7.51 (5.01, 11.25)
No	1.00	1.00	1.00	1.00
*Social/environmental factors*				
Ever lived on the streets (homelessness)				
Yes	1.80 (1.35, 2.40)	1.14 (0.80, 1.62)	1.76 (1.19, 2.60)	0.94 (0.59, 1.49)
No	1.00	1.00	1.00	1.00
Living in an unsafe neighborhood				
Yes	1.63 (1.22, 2.17)	1.29 (0.93, 1.79)	2.15 (1.47, 3.13)	1.62 (1.06, 2.48)
No	1.00	1.00	1.00	1.00

Note: Parental alcohol use was not used as a covariate in the analyses of alcohol-related physical abuse since that measure was used to establish the outcome variable.
